# Patent Term Extension for Innovative Drugs in China: A Cohort Study from 2021 to 2024

**DOI:** 10.1007/s43441-025-00903-x

**Published:** 2025-12-11

**Authors:** Xuefang Yao, Yan Zhang, Jin Zhou

**Affiliations:** School of Health Economics and Management, Nanjing University of Chinese Medicine, Nanjing, Jiangsu Province China

**Keywords:** Patent term extension, Effective patent life after listing, Innovative drug, Access to medicines, China

## Abstract

**Background:**

In January 2024, China officially implemented the patent term extension (PTE) system, that is, began to implement PTE for innovative drugs approved after June 1, 2021. This study analyzed the PTE system’s outcomes in China and its potential impact on drug accessibility.

**Methods:**

We conducted a simulation analysis of the PTE duration and effective patent life after listing (EPLL) of innovative drugs approved by Chinese regulatory authorities from June 2021 to December 2024, using data collected from public databases such as China’s marketed drug patent registration platform.

**Results:**

A total of 148 innovative drugs were included in the study. Among them, 61% were able to obtain PTE, with a median PTE duration of 5 years (IQR: 2.3–5). The median EPLL before adding PTE was 9 years (IQR: 6.1–11.7), and the median EPLL after adding PTE was 14 years (IQR: 11.1–14), an increase of 55.6%. Furthermore, the study reveals variations across drug types and therapeutic areas.

**Conclusions:**

Compared to findings from prior studies on the U.S. and the European Union, the median PTE duration for innovative drugs in China is longer, while the median EPLL is only marginally extended. Both Chinese domestic stakeholders and foreign enterprises should evaluate the potential impact by considering both the incremental PTE periods and the overall EPLL levels.

**Supplementary Information:**

The online version contains supplementary material available at 10.1007/s43441-025-00903-x.

## Introduction

Drug patent term extension (PTE), also known as patent term restoration (PTR), is a system that extends the relevant patent term to compensate for patent life lost to clinical testing and regulatory review of new drug [1, 2]. Normally, the protection period of a patent is 20 years from the date of application, while the R&D cycle of an innovative drug is usually over 10 years, which greatly shortens the effective patent term, resulting in a significant reduction in the exclusivity period of innovative drugs after listing [3]. To this end, the U.S. passed the Drug Price Competition and Patent Term Restoration Act in 1984, which first established this system [4]. Subsequently, Japan, the European Union, Australia, Israel, South Korea, Russia, Canada and other countries have also established this system, which has become a more mature international patent rule [5].

From the perspective of the sources of international law, the PTE does not belong to the provisions of the Agreement on Trade-Related Aspects of Intellectual Property Rights (TRIPS) [6, 7]. In January 2020, the China‒US Economic and Trade Agreement (2020) clearly stipulated the PTE provisions [8]. In October 2020, China’s newly revised Patent Law established the PTE system for the first time [9]. In December 2023, with the promulgation of the newly revised *Implementing Rules of Patent Law* and *Patent Examination Guidelines* [10, 11], China’s PTE system officially entered practical operation. The China National Intellectual Property Administration (CNIPA) began the examination and approval of PTE requests for innovative drugs and modified new drugs approved after June 1, 2021 on January 20, 2024 [12], and has not yet announced the results of the PTE approval.

We took the U.S. (the system is more mature) and the European Union (the calculation method is similar to China) as the comparison objects, and sorted out the similarities and differences of the systems of China, the U.S. and the European Union (see Supplementary file 1) [13–15]. The PTE calculation method in China is similar to that of the European Union, using a simple calculation model to compensate for the time spent on innovative drug research and review, while requiring the PTE not to exceed 5 years and the compensated effective patent life after listing (EPLL) not to exceed 14 years.

Drug patents that are given PTE are important bellwether indicators for generic drug market entry [16]. This study selected the innovative drugs approved after the implementation of China’s PTE system as the research samples, conducted a cohort study, simulated the calculation of extension period, and then analyzed the results of the PTE of innovative drugs in China and the possible impact on drug access through statistics and comparison.

## Methods

### Study Design

This study selected innovative drugs approved by the National Medical Products Administration (NMPA) after the implementation of the system on June 1, 2021 as research samples. Through data collection, we simulated and calculated the PTE duration and effective patent life after listing (EPLL) of these samples, conducted statistical comparisons to present characteristics of PTE for innovative drugs in China, and explored their possible impact on drug accessibility. Innovative drugs in this study refer to those not listed in China or abroad that are specifically class 1 applications in the registration classification of chemical drugs, biological products, and traditional Chinese medicines (TCM) stipulated in China’s Drug Registration Administration Measures. The estimated patents mainly include those registered on China’s patent information registration platform for marketed drugs. Since there may be multiple patents for one innovative drug at the same time, the patentee can only request for PTE for one of the them. Therefore, when an innovative drug registers multiple patents, we select its key product patent covering its active ingredient for calculation.

### Data Collection

The samples of innovative drug were sourced from the annual drug evaluation report for 2021–2024 [17–20] published by NMPA. Sample collection took place between June 1, 2021 and December 31, 2024. The approved information about these innovative drugs was obtained from both NMPA’s drug query database and CDE’s information disclosure database. The patent-related details of these innovative drugs are gathered from China’s patent registration platform for marketed drugs as well as China’s patent examination information query database. Additionally, some innovative drugs lacking registered patents have their patent details supplemented through publicly available medical insurance negotiation data provided by the National Healthcare Security Administration.

### Data Simulation and Analysis

Data simulation includes the calculation of PTE duration and EPLL. The PTE duration is computed using Formula 1, which takes the minimum value among ‘the PTE calculation value, 5 years, and the calculation value of the effective patent term after listing equals 14 years.’ If this minimum value is less than or equal to zero, it indicates that no PTE applies. EPLL is determined by subtracting the approval date of the innovative drug from its patent expiration date. The specific calculation formula can be found in Table 1.

**Table 1:** Simulation formulas of PTE and EPLL

Subject	Simulation formulas
PTE	Formula 1: PTE duration = Min (the PTE calculation value, 5 years, the calculation value of the effective patent term after listing equals 14 years), if PTE duration ≤ 0, there is no PTE Among them, the PTE calculation value = approval date of innovation drug -patent application date-5 years. the calculation value of the effective patent term after listing equals 14 years = the approval date of innovative drugs + 14 years-the original patent expiry date
EPLL	Formula 2: EPLL = the patent expiration date-the innovative drug approval date

The patent expiration date in the EPLL calculation, if the patent has no PTE, is the original patent expiration date; if the patent has PTE, it is the original expiry date of the patent + PTE duration

Data analysis involves both descriptive statistics and difference analysis. In this study, Microsoft Excel 2016 was employed for conducting descriptive statistics on sample innovative drugs’ characteristics as well as for simulating results related to PTE and EPLL. Additionally, a non-parametric rank sum test was utilized to assess differences in PTE across various types of innovative drugs (chemical, biologics, TCM) and therapeutic areas (oncology, infection, others), along with evaluating differences in EPLL before and after adding PTE. SPSS Statistics 22.0 was used for data analysis while Origin 2024 facilitated the creation of corresponding charts.

## Results

### Characteristics of the Included Innovative Drugs

The study included 148 innovative drugs approved by NMPA from June 1, 2021 to December 31, 2024, comprising of 71 chemical innovative drugs, 55 biological innovative drugs, and 22 TCM innovative drugs. Among the total number of innovative drugs, only 14 were first-in-class drugs. In terms of expedited approval processes, it is notable that the number of innovative drugs that received Priority review was the largest, reaching 54, followed by conditional approval, reaching 48. By contrast, only 27 Breakthrough therapy designation were approved. Additionally, due to the impact of the COVID-19 pandemic, special approvals were granted to 10 innovative drugs. In terms of therapeutic areas, oncology and infection were the most prominent, accounting for 38% and 18%, respectively. The other therapeutic areas had lower numbers, all in single digits. For further details, please see Table 2.

**Table 2:** Characteristics of innovative drugs approved by NMPA in China from 2021 to 2024

Characteristics	Chemical, N (%)	Biologics, N (%)	TCM, N (%)	Total
Number of drugs	71(48)	55(37)	22(15)	148
*Year (number of approved agents)*				
2021	18(46)	11(28)	10(26)	39
2022	11(52)	6(29)	4(19)	21
2023	19(47.5)	16(40)	5(12.5)	40
2024	23(47.9)	22(45.8)	3(6.3)	48
*Novelty*				
First-in-class	6(43)	8(57)	0(0)	14
*Expedited programs*				
Priority review	34(63)	19(35)	1(2)	54
Breakthrough therapy designation	13(48)	14(52)	0(0)	27
Conditional approval	21(44)	25(52)	2(4)	48
Special approval	4(40)	6(60)	0(0)	10
*MAH type*				
Domestic	59(45.7)	48(37.2)	22(17.1)	129
Foreign	12(63.2)	7(36.8)	0(0)	19
*Therapeutic area*				
Oncology	27(48)	28(50)	1(2)	56
Infection	16(62)	10(38)	0(0)	26
Endocrinology, diabetes, and metabolism	9(82)	2(18)	0(0)	11
Psychiatry	2(33)	0(0)	4(67)	6
Digestive system	4(67)	0(0)	2(33)	6
Dermatologicals	3(30)	4(40)	3(30)	10
Urinary system	1(33)	0(0)	2(67)	3
Respiratory system	0(0)	0(0)	1(100)	1
Cardiovascular system	1(100)	0(0)	0(0)	1
Nervous system	2(100)	2(0)	0(0)	4
Genital system	1(100)	0(0)	0(0)	1
Blood and blood forming organs	2(67)	1(33)	0(0)	3
Various	3(15)	7(35)	10(50)	20

### PTE Simulation for Innovative Drugs

As shown in Table 3, in the 148 innovative drug samples, 61% could obtain PTE through simulated calculations, with a median PTE duration of 5 years (IQR: 2.3–5). All PTE periods reached the maximum allowed, with 45 (49%) reaching the 5-year limit and 46 (51%) reaching the 14-year limit. Additionally, regarding whether the patent holder of the innovative drug had submitted a PTE application to CNIPA, there were still 21 (23%) innovative drugs that had not submitted applications.

**Table 3:** PTE simulation results of approved innovative drugs in China from 2021 to 2024

Characteristics	Chemical, N (%)	Biologics, N (%)	TCM, N (%)	Total
Number of drugs	71	55	22	148
No patent/ patent registration	2(3)	21(38)	12(55)	35(24)
No PTE	11(15)	10(18)	1(5)	22(15)
With PTE	58(82)	24(44)	9(41)	91(61)
0 < PTE ≤ 1 years	3(5)	4(17)	0(0)	7(8)
1 < PTE ≤ 3 years	14(24)	6(25)	2(22)	22(24)
3 < PTE < 5 years	11(19)	6(25)	0(0)	17(19)
PTE = 5 years	30(52)	8(33)	7(78)	45(49)
Years of PTE (median)	5	3.5	5	5
Whether a request for extension has been submitted	58	24	9	91
Yes	48(83)	17(71)	5(56)	70(77)
No	10(17)	7(29)	4(44)	21(23)

**Fig. 1:**
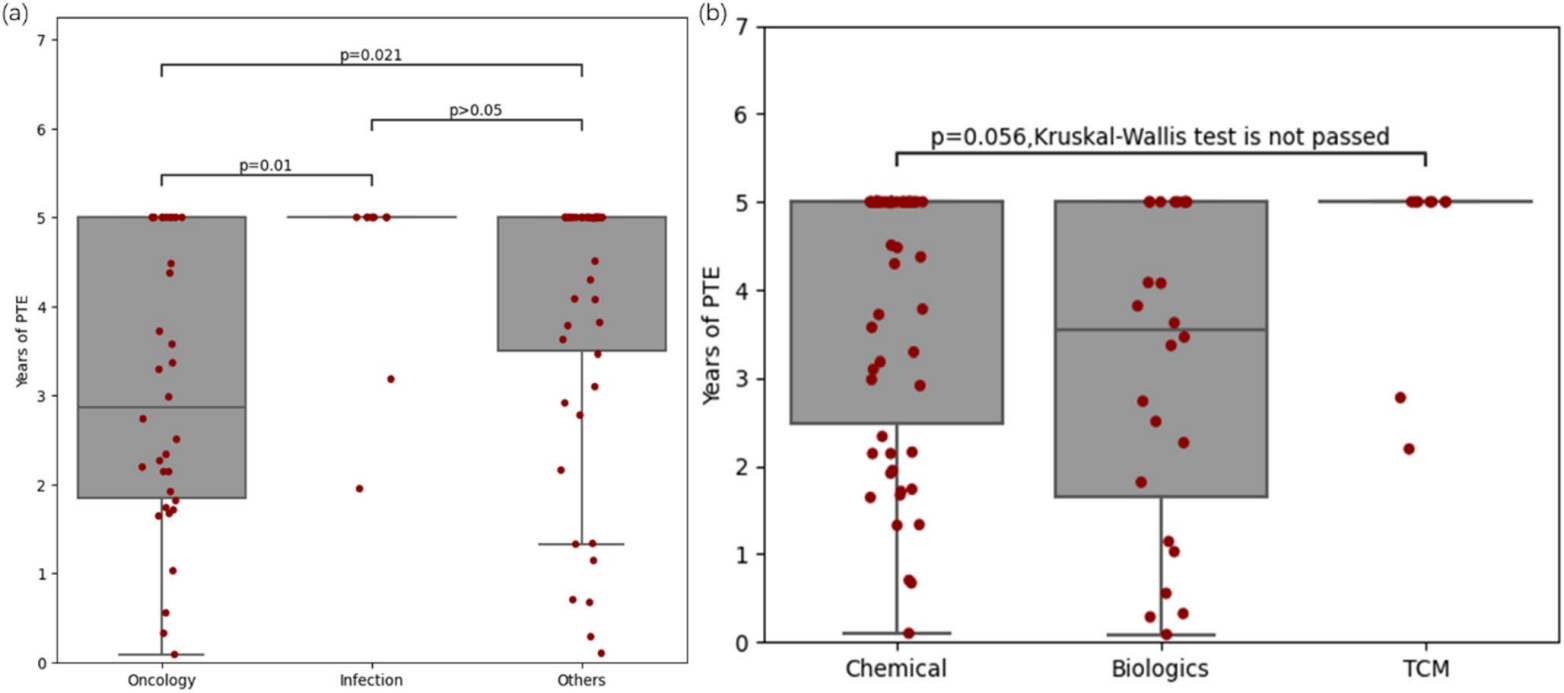
Comparison of the PTE of the innovative drugs in **a** three types and **b** three therapeutic areas. **a** Comparison of the PTE length of chemical, biologics, and TCM innovative drugs. **b** Comparison of the PTE length of innovative drugs in oncology, infection, others therapeutic areas. PTE, patent term extension; TCM, traditional Chinese medicine

In terms of the number of drugs that can obtain PTE, chemical innovative drugs had a clear advantage, reaching 58 (82%), while biological innovative drugs and TCM innovative drugs were less common, accounting for 24 and 9 (44% and 41%, respectively). Regarding the distribution of PTE duration, both chemical and biological innovative drugs showed distributions across all numerical segments. However, TCM innovative drugs were more concentrated with 78% having a PTE duration of 5 years. The median PTE duration for the three types of innovative drugs was 5 years for chemical innovative drugs, 5 years for TCM innovative drugs, and 3.5 years for biological innovative drugs. Additionally, as shown in Fig. 1b, there was no significant difference observed between the durations among these three types.

From the PTE characteristics in different therapeutic areas (Table 4), we found that the proportion of innovative drugs that can obtain PTE was highest in others (94%), followed by oncology and infection (71% and 69%, respectively). Regarding the distribution of PTE duration, there was relatively uniform distribution in the oncology area, while it was more concentrated in the infection area, with 82% obtaining a PTE duration of 5 years. Furthermore, significant differences were observed between oncology and infection (*P* = 0.01), as well as between oncology and others (*P* = 0.021), while no significant difference existed between infection and others (*P* > 0.05) (Fig. 1a). The median PTE duration of the three therapeutic areas was 2.9 years for oncology, 5 years for infection and 5 years for others, respectively.

**Table 4:** Characteristics of PTE obtained from innovative drugs in different therapeutic areas

Characteristics	Oncology, N (%)	Infection, N (%)	Others, N (%)
Number of drugs	56	26	66
With patent registration	48(86)	16(62)	49(74)
No PTE	14(29)	5(31)	3(6)
With PTE	34(71)	11(69)	46(94)
Years of PTE (median)	2.9	5.0	5.0
0 < PTE ≤ 1 years	4(12)	0(0)	4(9)
1 < PTE ≤ 3 years	14(41)	1(9)	6(13)
3 < PTE < 5 years	6(18)	1(9)	9(20)
PTE = 5 years	10(29)	9(82)	27(59)

### Effective Patent Life of Innovative Drugs After Listing

The effective patent life after the listing of an innovative drug (EPLL) basically determines the exclusivity period of the drug in the market, thereby affecting the entry time of generic drugs and the drug’s accessibility. As shown in Table 5, among the 91 innovative drugs that can obtain PTE, the median EPLL before adding PTE was 9 years (IQR: 6.1–11.7), and the median EPLL after adding PTE was 14 years (IQR: 11.1–14), an increase of 55.6%. After statistical analysis, the EPLL before adding PTE that reaches 10 years or more was only 43%, and the percentage after adding PTE reached 82%; the EPLL before adding PTE that was in the 13–14 years interval was only 9%, and the percentage of EPLL equal to 14 years after adding PTE reached 51%.

**Table 5:** The median EPLL of innovative drugs before and after adding PTE (years)

Characteristics	Without PTE	With PTE	*P* value
Number of drugs	91	91	
Years of EPLL (median)	9.0	14.0	< 0.01
*Drug types*			
Chemical	8.8	13.8	< 0.01
Biologics	10.5	14	< 0.01
TCM	4.7	9.7	< 0.01
*Therapeutic areas*			
Oncology	11.1	14.0	< 0.01
Infection	7.5	12.5	< 0.01
Others	8.2	13.2	< 0.01

Among the three types of innovative drugs, TCMs had a lower EPLL with a median value of 4.7 years before adding PTE and a median value of 9.7 years after adding PTE. In terms of therapeutic areas, oncology had a longer EPLL with a median value of 11.1 years before adding PTE and a median value of 14 years after adding PTE. Furthermore, from the analysis of variability, there were significant differences in EPLL length before and after adding PTE for all sample innovative drugs, for the three types of innovative drugs, and for the three therapeutic areas of innovative drugs.

## Discussion

### PTE and Market Exclusivity

The PTE system has been implemented in regions such as the U.S. and the European Union for over 30 years, and numerous studies have been conducted to assess its effectiveness. According to a study of the 170 top-selling drugs approved in the U.S. from 2000 to 2012, 49% (83 drugs) received a PTE, the median PTE duration was 2.75 years (IQR: 1.5–4.0). The addition of PTE extensions alone shifted the expiration dates of the patents from a median of 9.5 years (IQR: 6.75–11.75) after FDA approval to a median of 13.25 years (IQR: 10.25–14.0) after FDA approval, an increase of 40% [21]. Another study statistically analyzed 688 new drugs in the U.S. from 2000 to 2018, of which 319 (45%) received PTE, the median PTE duration was 2.3 years (IQR: 1.6–3.6). The median EPLL with PTE was 12.9 years (IQR: 10.5–14) [22]. Two simulation studies indicated that 88 (82%) of the 107 new chemical entities (NCEs) approved in the U.S. from 2018 to 2021 were able to obtain PTE, with a median PTE duration of 2.9 years, and the median EPLL after combining PTE duration was 13.4 years [23]. In addition, 47 (53%) of the 88 new biological products approved in the U.S. from 2015 to 2021 were able to obtain PTE, with a median PTE duration of 2.3 years (The proportion of those who obtained 5-year PTE was 24%), while 62 (64%) of the 98 new biological products approved in the European Union were able to obtain SPC, with a median SPC duration of 2.4 years (The proportion of those who obtained 5-year SPC was 37%). The median EPLL for new biological products with PTE/SPC in the U.S. and EU were 13.6 years and 12.8 years, respectively [24].

Since China has not yet truly implemented the regulatory data protection system, the market exclusivity of innovative drugs is mainly set by the key product patent, which usually covers its active ingredient. Therefore, it is generally believed that the implementation of PTE undoubtedly extends the market exclusivity period of innovative drugs, delays the entry of generic drugs, and consequently has a negative impact on drug accessibility. Since According to the simulation results of this study, among 148 samples of innovative drugs, 91 (61%) can obtain PTE with a median PTE duration of 5 years. Compared with the aforementioned literature, the median PTE duration in China is longer than that in both the U.S. and European Union (both ranging from 2 to 3 years). This implies that after PTE implementation the extension of China’s innovative drug market exclusivity period is more obvious and the impact on drug accessibility is also more obvious compared to those in the U.S. and European Union.

However, PTE is only an indicator of the increase. Normally, in the absence of patent invalidation, we determine the length of market exclusivity for an innovative drug based on the effective patent term, i.e., the time from the drug’s approval to patent expiry. Our simulation showed that, among the 91 innovative drugs for which PTE is available, the EPLL as a whole increased significantly after adding PTE, moving from a median average of 9 years (IQR: 6.1–11.7) to a median of 14 years (IQR: 11.1–14), and the proportion of EPLL exceeding 10 years increased from 43 to 82%. In addition, if we compare the EPLL results on an international level, we find that the median EPLL of Chinese innovative drugs (14 years) is also slightly higher than that of the U.S. (about 13 years) and the European Union (12.8 years) in the above literature statistics. However, we also found that the difference of EPLL (about 1 year) is smaller than that of PTE (2–3 years). The reason for this difference may be attributed to longer R&D duration for innovative drugs in China than in the U.S. and the European Union. Because PTE is a compensation for the time-consuming development and review of new drugs, but the compensation period should not exceed 5 years, and the proportion of China’s innovative drug PTE equal to 5 years is 49%, which indicates that half of innovative drug development and review time is longer than 5 years, so after adding PTE, the difference between its EPLL and the U.S. or the European Union has indeed narrowed.

### PTE of Different Innovative Drugs

The R&D of innovative drugs varies depending on the types of drugs and the therapeutic areas, and the PTE is directly related to the R&D of innovative drugs. In terms of the number of drugs that can obtain PTE, chemical innovative drugs have a significant advantage, reaching 58 (82%), while biological innovative drugs and TCM innovative drugs are fewer, being 24 and 9 (respectively accounting for 44% and 41%). This may be related to the fact that biologics and TCM have not yet established complete patent linkage, thereby leading to a low patent registration willingness. In terms of the PTE duration, the median PTE of TCM innovative drugs and chemical innovative drugs is 5 years, with 78% of TCM innovative drugs having a PTE of 5 years, while the median value of biological innovative drugs is only 3.5 years. This shows that from the perspective of increasing the market exclusivity period through PTE, TCM innovative drugs benefit the most, while biological innovative drugs benefit the least. However, it is interesting that after adding PTE, the median EPLL in the three types of innovative drugs is in the opposite order, with biological innovative drugs being the longest (14 years), followed by chemical innovative drugs (13.8 years), and TCM innovative drugs being the shortest (9.7 years). This contrast reflects the difference in the R&D time of three types of innovative drugs in China, the R&D speed of biological innovative drugs is relatively fast, and the R&D speed of TCM innovative drugs is relatively slow. From the perspective of the remaining market exclusivity period after the innovative drug is approved, biological innovative drugs and chemical innovative drugs undoubtedly lead TCM innovative drugs.

The statistical analysis of the three therapeutic areas shows that in terms of PTE acquisition, others have the highest rate (94%), followed by oncology and infection, which are 71% and 69% respectively, indicating that around 30% of innovative drug patents in the fields of oncology and infection have failed to obtain PTE. The main reason is that the remaining effective life of the original patent has exceeded 14 years. In terms of PTE duration, infection and others have longer median values (both 5 years), while oncology has a shorter median value (2.9 years), and there are significant differences between oncology and infection and others, indicating that from the perspective of PTE duration, the benefits of innovative drugs in the oncology field are relatively small. However, the ranking changes again when the median EPLL after adding PTE is considered, with oncology ranked first (14 years), followed by others (13.2 years) and infection (12.5 years). This change also reflects that the R&D speed of innovative drugs in the oncology field is relatively the fastest, and its market exclusivity after listing is the longest.

### Study Limitations

This study has several limitations. First, since the China’s PTE system has been implemented for a short time, most of the PTE applications for innovative drugs are still under review and the results have not been announced yet. Therefore, in this study, the PTE duration for the sample of innovative drugs that meet the conditions is calculated using a simulation method and the data results are discussed. However, there may be some discrepancies between the simulated results of this study and the final implementation results. Second, in the discussion of comparisons with the implementation results in the U.S. and the European Union, we used literature data. If a contemporaneous cohort study can be conducted in the future, the discussion will be more persuasive. We will continue to pay attention to the implementation results of China’s PTE system and the comparative analysis with other countries’ contemporaneous cohort studies in the future.

## Conclusion

The PTE duration and EPLL can present the impact of the PTE system on the market exclusivity and drug accessibility from different dimensions. From the perspective of the overall impact of drug accessibility, this study shows that the median PTE duration for Chinese innovative drugs is longer than that in the U.S. and the European Union, but the median EPLL after adding PTE is slightly longer than that in U.S. and European Union. The main reason for this difference is that the average R&D speed of innovative drugs in China is slightly lower than that of the U.S. and the European Union. From the perspective of the benefits of different innovative drugs, according to the median PTE duration for the three types of innovative drugs, TCM innovative drugs have the greatest benefit, but when adding PTE, the median EPLL ranking is reversed, in order of biological innovative drugs, chemical innovative drugs, and TCM innovative drugs. From the median PTE duration for the three therapeutic areas, the duration of infection and others is the longest, but when adding PTE, the median EPLL ranking changes again, in order of oncology, others, and infection. In addition, foreign innovative drug companies should note that if they want to obtain PTE in China, they must list China as one of the first countries to launch their innovative drugs, otherwise they will lose the opportunity to obtain PTE because they do not belong to the definition of innovative drugs in China. Chinese domestic stakeholders and foreign enterprises should assess its possible impact by taking into account both incremental PTE periods and overall levels of EPLL.

## Supplementary Information

Below is the link to the electronic supplementary material.

Supplementary Material 1

## Data Availability

No datasets were generated or analysed during the current study.

## Abbreviations

CNIPA: China National Intellectual Property Administration
CDE: Center for Drug Evaluation
EPLL: Effective patent life after listing
FDA: U.S. Food and Drug Administration
MAH: Marketing authorization holder
NMPA: National Medical Products Administration
NCE: New chemical entity
PTE: Patent term extension
TCM: Traditional Chinese medicine
