# Supplementary material for: Patent Term Extension for Innovative Drugs in China: A Cohort Study from 2021 to 2024
Source: Ther Innov Regul Sci. 2025 Dec 11;60(2):544–50. doi: 10.1007/s43441-025-00903-x (PMC12945983; doi:10.1007/s43441-025-00903-x)
Supplement: Supplementary file 1 — Supplementary Material 1 [file 43441_2025_903_MOESM1_ESM.docx]

**Supplementary file 1**

**Table S1** Comparison of patent term extension systems in China, the U.S. and the European Union

|  | **China** | **the US** | **the EU** |
| --- | --- | --- | --- |
| **Name of system** | Patent term restoration (PTR) | Patent term extension (PTE) | Supplementary protection certificate (SPC) |
| **Competent department** | China national intellectual property administration (CNIPA) | United states patent and trademark office (USPTO) | National patent offices (NPOs) |
| **Scope of applicable drugs** | Innovative drugs, some improved new drugs | Drugs containing a new active ingredient approved for the first time, including salts, esters, or new formulations of the active ingredient. | Any substance or composition of substances used to treat or prevent disease in humans or animals, as well as pediatric drugs |
| **Patent type** | Product patent, process patent, use patent | Product patent, process patent, use patent | Product patent, process patent, use patent |
| **Calculation method of extension term** | PTR calculation value = innovation drug approval date－patent application date－5 years | PTE calculation value = 1/2 the duration between IND approval and NDA submission + the duration between NDA submission and NDA approval－the length of due diligence | SPC calculation value = date of first marketing approval in the EU or Member State－patent application date－5 years |
| **Limitation of extension term** | Extension term up to a maximum of 5 years;  The EPLL of the drug patent after extension must not exceed 14 years | Extension term up to a maximum of 5 years;  The EPLL of the drug patent after extension must not exceed 14 years | Extension term up to a maximum of 5 years;  The EPLL of the drug patent after extension must not exceed 15 years |
| **Other requirements** | (1) The patent granted date shall be earlier than the drug approval date  (2) The patent is valid  (3) The patent term has not been extended  (4) Only one patent term extension per drug is allowed  (5) A patent can only be extended once  (6) During the extension period, the scope of protection of the patent is limited to the new drug approved for market, and is limited to the approved indication of the new drug | (1) The patent has not expired before the application for extension is filed  (2) The patent term has not been extended  (3) Only one patent term extension per drug is allowed  (4) A patent can only be extended once  (5) During the extension period, the scope of protection of the patent is limited to the indication approved by the FDA, and the scope of protection of the preparation method patent is limited to the approved drug preparation step | (1) The patent is valid  (2) The patent term has not been extended  (3) Only one patent term extension per drug is allowed  (4) A patent can only be extended once  (5) Pediatric medication can be extended by 6 months, and the maximum duration of SPC is 5.5 years |

Note：

EPLL: Effective patent life after listing; IND: Investigational new drug; NDA: New drug application; PTE: Patent term extension; PTR: Patent term restoration; SPC: Supplementary protection certificate.
